# Replacing Myalgic Encephalomyelitis and Chronic Fatigue Syndrome with Systemic Exercise Intolerance Disease Is Not the Way forward

**DOI:** 10.3390/diagnostics6010010

**Published:** 2016-02-05

**Authors:** Frank N.M. Twisk

**Affiliations:** ME-de-patiënten Foundation, Zonnedauw 15, 1906 HB Limmen, The Netherlands; frank.twisk@hetnet.nl; Tel.: +31-72-505-4775

**Keywords:** myalgic encephalomyelitis, chronic fatigue syndrome, systemic exercise intolerance disease, diagnosis, assessment

## Abstract

Myalgic encephalomyelitis (ME), described in the medical literature since 1938, is characterized by distinctive muscular symptoms, neurological symptoms, and signs of circulatory impairment. The only mandatory feature of chronic fatigue syndrome (CFS), introduced in 1988 and redefined in 1994, is chronic fatigue, which should be accompanied by at least four or more out of eight “additional” symptoms. The use of the abstract, polythetic criteria of CFS, which define a heterogeneous patient population, and self-report has hampered both scientific progress and accurate diagnosis. To resolve the “diagnostic impasse” the Institute of Medicine proposes that a new clinical entity, systemic exercise intolerance disease (SEID), should replace the clinical entities ME and CFS. However, adopting SEID and its defining symptoms, does not resolve methodological and diagnostic issues. Firstly, a new diagnostic entity cannot replace two distinct, partially overlapping, clinical entities such as ME and CFS. Secondly, due to the nature of the diagnostic criteria, the employment of self-report, and the lack of criteria to exclude patients with other conditions, the SEID criteria seem to select an even more heterogeneous patient population, causing additional diagnostic confusion. This article discusses methodological and diagnostic issues related to SEID and proposes a methodological solution for the current “diagnostic impasse”.

## 1. Introduction

**Figure 1:**
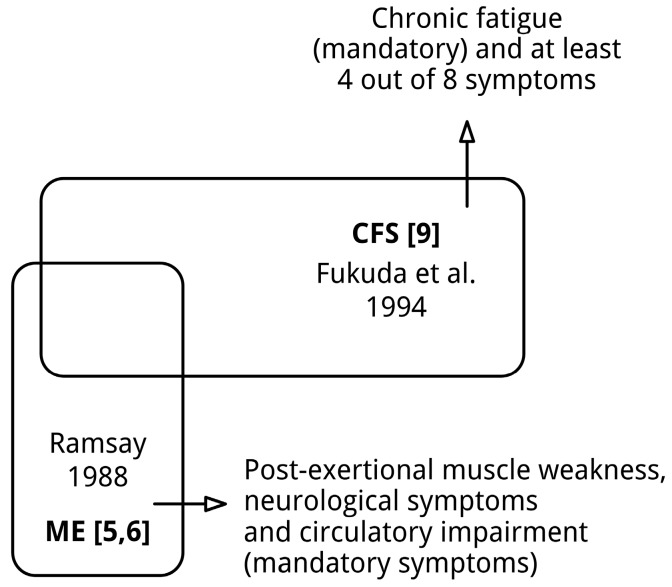
Myalgic encephalomyelitis (ME) and chronic fatigue syndrome (CFS): two distinct, partially overlapping diagnoses. The sizes of the shapes do not reflect the absolute sizes of various patient (sub)populations.

In 1938 a detailed analysis of an outbreak of “atypical poliomyelitis” among the personnel of the Los Angeles County General Hospital during the summer of 1934 was published [1]. Since then, myalgic encephalomyelitis (ME) has been described under various names, mainly on account of outbreaks [2,3], and in 1956 ME was identified as a new clinical entity [4] in response to an outbreak in the Royal Free Hospital in London in 1955 [5] and earlier outbreaks all over the world. Based upon an analysis of the literature until then, the clinical picture of ME was described by Ramsay *et al.* [5,6] in the late 1980s. ME is primarily defined by distinctive neuro-muscular symptoms: prolonged muscle weakness after minor exertion, neurological symptoms indicative of cerebral dysfunction, and circulatory impairment, and a chronic relapsing course. Much of the confusion with regard to ME originates from the introduction of the diagnostic entity chronic fatigue syndrome (CFS) [7] by the US Centers for Disease Control and Prevention (CDC) in 1988 [8]. CFS was redefined in 1994 [9]. The only mandatory symptom of CFS is chronic fatigue, which should be accompanied by four out of eight “additional” symptoms,” e.g., unrefreshing sleep and headaches. Since then the focus of research shifted from ME to CFS. This introduced two major methodological problems. Firstly, the diagnostic criteria for ME and CFS define two distinct, partially overlapping, clinical entities (see Figure 1). For example, fatigue is not required for the diagnosis ME, while post-exertional muscle weakness and typical neurological symptoms are not required to meet the diagnosis CFS. Secondly, due to its polythetic nature, the CFS [9] criteria define a heterogeneous group of people with chronic fatigue [10,11,12]. Not surprisingly, research into CFS has often yielded contradictory results or abnormalities present in subgroups of patients [13].

In order to resolve the diagnostic confusion, partly due to the introduction of CFS in the 1980s/1990s [8,9], the Institute of Medicine (IOM) was asked “to define diagnostic criteria for myalgic encephalomyelitis/chronic fatigue syndrome, to propose a process for the reevaluation of these criteria in the future, and to consider whether a new name for this disease is warranted” [14] (p. xv). In response to that request, the IOM proposed that a new clinical entity, systemic exercise intolerance disease (SEID), defined by new diagnostic criteria, should replace the clinical entities ME and CFS. This clinical entity SEID has already been embraced by some researchers [15]. This article discusses the shortcomings of the method by which SEID was developed and its outcome, the diagnostic criteria of SEID, and proposes a methodological solution for the current diagnostic impasse regarding ME and CFS.

## 2. Methodological Shortcomings of the Development Procedure of Systemic Exercise Intolerance Disease (SEID)

This paragraph discusses implications of the starting points of the development process, which significantly affected the outcome, which will be discussed in the next paragraph.

### 2.1. The Pre-Assumption that Myalgic Encephalomyelitis (ME) and Chronic Fatigue Syndrome (CFS) Denote “Similar Conditions” Is Invalid

The IOM consider ME and CFS to be “conditions with similar symptoms” [14] (p. 1). According to the IOM, “Many patients prefer ‘myalgic encephalomyelitis’”, because “they believe it better reflects the medical nature of the illness” [14] (pp. 30–31). As the IOM [14] report notes: “[T]here are patients and researchers who maintain that ME and CFS are two different illnesses and oppose simply changing the name of CFS to ME [13].” [14] (p. 31). However, the position that ME and CFS are distinct, partially overlapping conditions, is not a matter of opinion, but a matter of definition. As can be seen in List 1, typical neuro-muscular symptoms define ME [5,6], while the only mandatory feature of CFS [9] is [unexplained] chronic fatigue (List 2). Despite overlap, muscular and neurological features typical for ME are not required to fulfill the case criteria for CFS, while “fatigue” is not essential for ME. List 3 illustrates a patient fulfilling the diagnosis CFS with no characteristic ME symptom at all, while List 4 depicts the clinical picture of an ME patient not meeting the diagnosis CFS.

**List 1.** The original diagnostic criteria of ME [5,6].

Distinct features of ME are:

- A unique form of muscle fatiguability: prolonged muscle weakness (and myalgia), lasting for days, even after a minor degree of physical effort (*^1^).
- Circulatory impairment, implicated by cold extremities and hypersensitivity to climatic change, but above all an ashen-grey facial pallor approximately 20 or 30 min before the patient complains of feeling ill.
- Cerebral dysfunction: impairment of memory and concentration and emotional lability, alterations of sleep rhythm (*^2^), vivid dreams (*^2^), episodic sweating and orthostatic tachycardia as cardinal features (the latter two not always present).
- Variability and fluctuation of both symptoms and physical findings over the day.
- A tendency to become chronic.

(*^1^) While post-exertional “malaise” (PEM), defined as an exacerbation of symptoms after physical or cognitive exertion or orthostatic stress, is an element of the diagnostic criteria of CFS, the ME criteria specifically require prolonged post-exertional muscle weakness (and muscle pain) after a minor physical effort. (*^2^) Although both ME and CFS symptoms relate to sleep, reversal of sleep rhythm and unrefreshing sleep are different types of symptoms.

**List 2.** The diagnostic criteria of CFS [9].

- Severe chronic_fatigue for 6 or more consecutive months, that is not due to ongoing exertion or other medical conditions associated with fatigue and significantly interferes with daily activities and work,
- Accompanied by at least 4 or more of the following 8 symptoms:
  - post-exertional malaise lasting more than 24 h (*^1^);
  - unrefreshing sleep (*^2^);
  - significant impairment of short-term memory or concentration;
  - muscle pain;
  - joint pain without swelling or redness;
  - headaches of a new type, pattern, or severity;
  - tender lymph nodes in the neck or armpit; and
  - a sore throat that is frequent or recurring.

(*^1^) While post-exertional “malaise” (PEM), defined as an exacerbation of symptoms after physical or cognitive exertion or orthostatic stress, is an element of the diagnostic criteria of CFS, the ME criteria specifically require prolonged post-exertional muscle weakness (and muscle pain) after a minor physical effort. (*^2^) Although both ME and CFS symptoms relate to sleep, reversal of sleep rhythm and unrefreshing sleep are different types of symptoms.

**List 3.** Example of a patient with CFS [9] not fulfilling the diagnosis ME [5,6].

Clinical picture of a patient fulfilling the diagnosis CFS:

- chronic fatigue for 6 or more consecutive months;
- unrefreshing sleep;
- significant impairment of short-term memory or concentration;
- headaches of a new type, pattern, or severity; and
- a sore throat that is frequent or recurring.

**List 4.** Example of a patient with distinctive ME [5,6] symptoms not meeting the diagnosis CFS.

Clinical picture of a patient fulfilling the diagnosis criteria of ME:

- prolonged muscle weakness and muscle pain after minimal exertion;
- circulatory impairment, e.g., indicated by cold extremities, disturbed thermoregulation, low body temperature, and orthostatic tachycardia; and
- cognitive impairment and other symptoms indicating neurological dysfunction.

### 2.2. The Literature Analyzed by the Medicine (IOM) Committee Largely Relates to CFS Research

Scientific literature from 1938 [1] until the late 1980s relate to findings in ME, as defined in 1988 [5,6], while almost all research studies in the last decades relate to symptoms and abnormalities in CFS, defined in 1988 [8] and redefined in 1994 [9]. Research into CFS, thoroughly analyzed research by the IOM [14], is not applicable to ME, since only a part of the patient population potentially meets the diagnostic criteria for ME, while not all ME patients qualify as CFS patients. Likewise, research into ME, which got far less attention in the analysis, cannot be generalized to CFS.

### 2.3. Consensus on “an Unclear Picture of the Symptoms” in a Heterogeneous Patient Group Does Not Guarantee a Good Solution

The IOM performed a comprehensive review of studies of (a) fatigue; and (b) notions related to the “minor” symptoms of CFS [9]: post-exertional “malaise” (an exacerbation of symptoms after physical or cognitive exertion), neurocognitive manifestations (relating to impairment of short-term memory or concentration), sleep (relating to unrefreshing sleep), pain (relating to muscle pain, multi-joint pain and headaches: three “minor” symptoms of CFS), and immune manifestations (relating to tender lymph nodes and sore throat); and/or (c) findings associated with autonomic and neuroendocrine manifestations and infections in ME and/or CFS. Articles, citations of the articles and “grey” literature were evaluated by two to five committee members assigned to each topic using a modified “GRADE grid” [16,17], after which a recommendation was made based upon consensus in the committee. In addition to the fact that the IOM committee largely based their opinion of “ME/CFS” on research into CFS, this working method introduces three additional issues: (1) the current definition of CFS [9] is leading, therefore typical ME symptoms, e.g., prolonged post-exertional muscle weakness, circulatory impairment, and specific symptoms related to cerebral dysfunction, were not taken into consideration; (2) some candidate symptoms were included in the analysis arbitrarily, e.g., autonomic dysfunction, while other possible symptoms, e.g., visual symptoms, were not analyzed; and (3) for a number of reasons, e.g., expert panel composition [18] and low interrater reliability [19], consensus on “an unclear picture of the symptoms and signs” [14] (p. 72) in heterogeneous patient samples may lead to arbitrary decisions. This latter issue is exemplified by the important role of orthostatic intolerance in the diagnosis SEID, a less common symptom in CFS [20], while more prevalent symptoms of CFS [20], e.g., muscle pain and flu-like symptoms, are arbitrarily not included in the definitional criteria of SEID [14].

## 3. Diagnostic Shortcomings of the New Definition for “ME/CFS”: SEID

According to the IOM [14], a diagnosis of SEID should be made if three symptoms are present and if the patient reports at least one of two facultative symptoms (List 5).

Looking at the definition of SEID three important observations can be made. Firstly, although ill-defined, post-exertional “malaise” is mandatory for the diagnosis SEID. Secondly, chronic fatigue, a vague and ambiguous concept that has created much confusion, still has a central role in the diagnostic criteria. Lastly, all core symptoms are abstract and should be assessed using questionnaires and patient self-report.

**List 5.** Proposed diagnostic criteria for SEID [14] (p. 6).

The diagnosis (SEID) requires that the patient have the following three symptoms:

- a substantial reduction or impairment in the ability to engage in pre-illness levels of occupational, educational, social, or personal activities, that persists for more than 6 months and is accompanied by fatigue, which is often profound, is of new or definite onset (not lifelong), is not the result of ongoing excessive exertion, and is not substantially alleviated by rest;
- post-exertional malaise (*^1^); and
- unrefreshing sleep (*^1^)

At least one of the two following manifestations is also required:

- cognitive impairment (*^1^); or
- orthostatic intolerance

(*^1^) Frequency and severity of symptoms should be assessed. The diagnosis (SEID) should be questioned if patients do not have these symptoms at least half of the time with moderate, substantial, or severe intensity.

### 3.1. Neither ME nor CFS Is Covered by the Diagnostic Criteria of SEID

**Figure 2:**
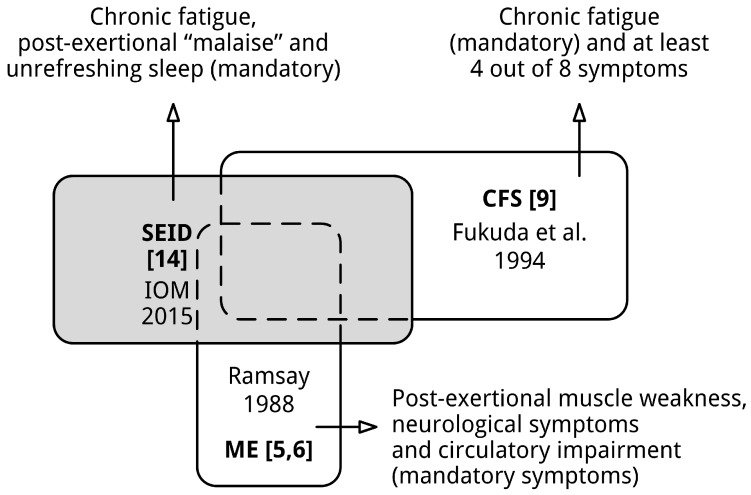
SEID is neither covering ME nor CFS. The sizes of the shapes do not reflect the absolute sizes of various patient (sub)populations.

Both ME and CFS are not covered by the proposed diagnostic criteria for SEID [14]. Firstly, the clinical entity SEID does not capture the essence of ME (List 1). Hallmark symptoms of ME, long-lasting post-exertional muscle weakness, neurological dysfunction, e.g., implicated by cognitive impairment and hyperacusis, and symptoms related to circulatory impairment, are not required to meet the diagnostic criteria of SEID. The huge discrepancy between SEID and ME is largely due to two methodological shortcomings discussed in the previous section: The premise that ME and CFS are similar conditions is invalid and the development process was almost solely based upon research into CFS patient populations. Secondly, although fatigue is also a principle feature of SEID, SEID does not include facultative symptoms of CFS [9], often present in patients, e.g., muscle pain and flu-like feelings [20], while orthostatic intolerance not incorporated in the diagnosis criteria for CFS [9] has been given a prominent role in the diagnosis [14]. While a few studies observed high prevalence rates for orthostatic intolerance in CFS [21], several studies have found much lower prevalence rates of orthostatic intolerance in CFS [22,23,24]. Looking at the high prevalence of symptoms left out of the diagnostic criteria, e.g., muscle pain, including this symptom seems odd. Since subgroups of patients with orthostatic intolerance also report fatigue, unrefreshing sleep and exercise intolerance, these patients could also meet the diagnosis SEID. All in all, SEID cannot adequately replace the diagnostic entities ME and CFS. The position of SEID [14] in relation to ME [5,6] and CFS [9] is illustrated by Figure 2.

### 3.2. The Abstract and Ill-Defined Symptoms of SEID Cannot Be Assessed by Self-Report

ME and CFS are clinical entities defined by their symptoms. However, characteristic symptoms of ME, e.g., post-exertional muscle weakness, and CFS, e.g., fatigue and unrefreshing sleep, are subjective and can neither be measured nor compared. For example, Jason *et al.* [25] found that “fatigue” in ME/CFS has at least five dimensions (post-exertional exhaustion, wired: over-stimulated when very tired, brain fog: cognitive impairment, complete lack of energy, and flu-like fatigue/feelings) and that fatigue in ME/CFS is not the “fatigue” as experienced by the general population. Research in and diagnosis of ME and CFS is often based upon questionnaires and self-report of typical symptoms, e.g., using a visual analogue scale. However, reliability and validity, criteria to substantiate the legitimacy of a subjective outcome, do not ensure an adequate measure [26]. Even more, symptoms reported by patients, e.g., fatigue, do not have to correlate with objective measures of disability in ME/CFS. This is exemplified by the observation that patients with “chronic, but stable CFS” have a significantly decreased aerobic capacity, which is correlated with self-reported physical activity, but is not correlated with self-reported fatigue [27] and the finding that a reduction of “fatigue” is not reflected by increased physical activity levels [28]. The five symptoms defining the clinical entity SEID are ambiguous and subjective. For that reason assessing symptoms by questionnaires and scores based on self-report is insufficient. Introducing thresholds for frequency (at least half of the time) and severity (moderate, substantial, or very severe), as proposed by the IOM, does not resolve the issue of false negatives and false positives. Jason *et al.* [20], for example, observed that even when these thresholds are applied, 4.7% of the healthy controls still met the diagnostic criteria for CFS [9] and at least 4% of the patients would not meet the diagnosis of CFS, since their “fatigue” would be insufficient.

Considering the controversy [29], there is an ambiguity of subjective measures and there are opposing views on the nature of the symptoms: With, on the one hand, “unhelpful cognitions and behavior perpetuating the symptoms” [30,31] *versus*, on the other hand, distinctive biological abnormalities explaining a multisystem illness [32,33], it is essential that symptoms are assessed objectively wherever possible, and not by subjective measures from self-report only. As the IOM report [14] states: “(Twisk) asserts that (…) objective assessment must address the unique symptoms in accordance with the diagnosis, whether it be ME or CFS” [34]. Various symptoms of ME and CFS can be assessed objectively, e.g., cognitive impairment, (post-exertional) muscle weakness, post-exertional “malaise” (long-lasting negative impact of exertion on symptoms) and orthostatic intolerance [35].

### 3.3. The SEID Criteria Do Not Seem Reduce the Heterogeneity of the CFS Patient Population

There is ample evidence that the diagnostic criteria for CFS define a heterogeneous population of people with chronic fatigue [10,11,12]. Introducing a new diagnostic entity, SEID, should have helped to resolve this issue. However, Jason *et al.* [36] recently found that the SEID criteria [14] are not substantially more restrictive than the CFS criteria [9] in a selective group of people with a self-reported diagnosis of ME and/or CFS. While 92% of the patients fulfilled the diagnosis of CFS [9], 88% met the SEID criteria. Therefore, the new criteria do not seem to reduce the diversity of the patient population. Even more, reducing the number of symptoms required and leaving out very common CFS symptoms, (e.g., muscle pain and flu-like feeling), will most likely increase the heterogeneity. It is not known how many people would meet the diagnosis SEID [14] without fulfilling the CFS criteria [10] in a non-selective group of people from the general population.

### 3.4. The Definition of SEID Includes People with Other Conditions

**Figure 3:**
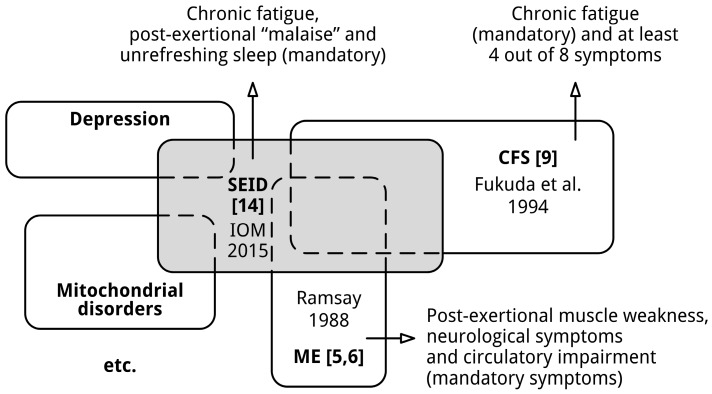
SEID overlaps with other (medical and psychiatric) conditions. The sizes of the shapes do not reflect the absolute sizes of various patient (sub)populations.

Contrary to the CFS definition [9], the SEID definition [14] does not exclude other disorders, so people with other medical and psychiatric conditions could meet the diagnosis SEID. As explained in the literature [37], people with other conditions also experience the symptoms, *i.e.*, fatigue, exercise intolerance, unrefreshing sleep, and orthostatic intolerance or cognitive impairment, essential to meet the diagnosis of SEID. Patients with any of the following conditions will all meet the criteria for a diagnosis of SEID: postural orthostatic tachycardia syndrome, chronic heart failure, chronic obstructive pulmonary disease, mitochondrial diseases, Addison’s disease, fibromyalgia and depression. Depression and medical conditions that may explain chronic fatigue are exclusionary criteria for the diagnosis CFS [9]. Not surprisingly, a recent study [38] found that substantial subgroups of patients with multiple sclerosis, lupus, and chronic fatigue who did not meet the diagnosis criteria of CFS [9] and had major depressive disorder fulfilled the diagnostic criteria for SEID [14]. The situation regarding the inclusion of other conditions is illustrated in Figure 3.

## 4. Proposal for a Methodological Solution for the Current “Diagnostic Impasse”

To resolve the “diagnostic impasse,” which for an essential part originates from the introduction of the ill-defined entity CFS [9], and to avoid additional confusion by introducing a new, only partially overlapping, clinical entity, SEID, it is crucial to develop empiric definitions for ME and conditions currently covered by the CFS “waste basket” diagnosis based upon the following methodological principles: (1) the original diagnostic criteria of ME [5,6] and criteria for CFS [9] define two, partially overlapping, conditions and should not be merged into a “hybrid”; (2) symptoms should be assessed by objective measures, not by self-report (only); (3) confounding variables, for example mode of onset, phase, duration of illness, gender, age and comorbidities, should be taken into account; (4) pattern recognition methods should be used instead of consensus; and (5) diagnostic labels should preferably reflect the clinical picture.

### 4.1. Make a Clear Distinction between Patients Meeting the Diagnosis of ME or CFS

As can be seen in Figure 1, ME [5,6]/CFS [9] is a “hybrid” not one diagnostic entity. It should also be noted that the diagnostic criteria of CFS define a diffuse syndrome, not a disease. To resolve the issue, patients meeting the (original) criteria [5,6] for ME, a distinct clinical entity [2], should be explored in detail, while the (remaining) patients fulfilling the diagnostic criteria for CFS [9] should also be analyzed in depth. Considering the heterogeneity of the CFS patient population, the (remaining) CFS patients most likely suffer from various disorders. Lumping patients (ME patients and CFS patients with various disorders) will not improve an accurate diagnosis. This is not only important for research purposes, but also for the clinical practice.

### 4.2. Symptoms Should Be Assessed Objectively If Feasible, Not Only by Self-Report

Considering the controversy with regard to the etiology and psychophysiology of ME and CFS [29,39] and the abstract and subjective nature of typical symptoms of ME and CFS, a diagnosis based upon self-reported measures is not adequate. As far is possible, objective methods should be employed to assess the symptoms and disability [35]. Characteristic symptoms, e.g., “fatigue,” long-lasting post-exertional weakness, cognitive impairment, post-exertional malaise (the negative effect of exercise on cognitive and physical performance levels) and visual problems can be “objectified” by using well-accepted methods, e.g., (repeated) cardiopulmonary exercise tests, neuropsychological tests (before and after an exercise test), (repeated) muscle power strength and endurance tests, tilt-table tests, and visual field tests. The status and disability of a patient should also be expressed in objective measures, e.g., activity levels, employment status and health care usage, as much as possible.

### 4.3. Take into Account Confounding Factors

Several findings show that the mode of onset, phase, duration of illness, gender, age and comorbidities, e.g., gastro-intestinal symptoms and (secondary) depression, can have an important influence on the clinical status and abnormalities in ME/CFS. The impact of these factors is illustrated by a study [40] that found clear gender differences in symptomology and a study [41] that established that sudden *vs.* gradual onset of CFS differentiates patients on cognitive and psychiatric measures. The influence of these variables on abnormalities is exemplified by a study [42] that found that patients with short-duration illness (≤3 years) exhibited pronounced activation of pro- and anti-inflammatory cytokines and dysregulation of intercytokine regulatory networks, while patients with long-duration illness showed an inverse picture, possibly due to “immune exhaustion” and a study [43] which observed elevated interleukin (IL)-8 levels in the spinal fluid of patients with sudden (viral-like) onset compared to patients with gradual onset and healthy controls.

### 4.4. Use Pattern Recognition Methods to Develop Empiric Definitions for ME, CFS-1 etc.

Based on the mandatory symptoms of ME (prolonged post-exertional muscle weakness, signs indicating cerebral dysfunction and symptoms reflecting circulatory deficits), pattern recognition methods could be employed to objectively establish optional symptoms of ME [5,6] and their prevalence rates, taken into account confounding factors. All symptoms should be assessed objectively wherever possible. Biomarkers should be investigated to validate the diagnosis ME biomarkers. Pattern recognition algorithms should also be used to subdivide the remaining population of patients fulfilling the current CFS [9] criteria and not meeting the ME criteria [5,6] in distinct CFS “symptom clusters” based upon mandatory symptoms and to establish optional symptoms in these symptom clusters” (CFS-1, CFS-2, *etc.*). Some researchers suggest that CFS patients with postural orthostatic tachycardia syndrome reflect a distinct patient subgroup of the CFS [9] population [22] with specific phenotypic features [23]. Pattern recognition methods could be used to reject or validate this position and to reveal other distinct patient populations. Interestingly, research has uncovered at least seven gene expression subtypes in CFS patients [44,45] with distinct differences in clinical phenotypes and severity.

### 4.5. Diagnostic Labels Should Preferably Reflect the Clinical Picture

**Figure 4:**
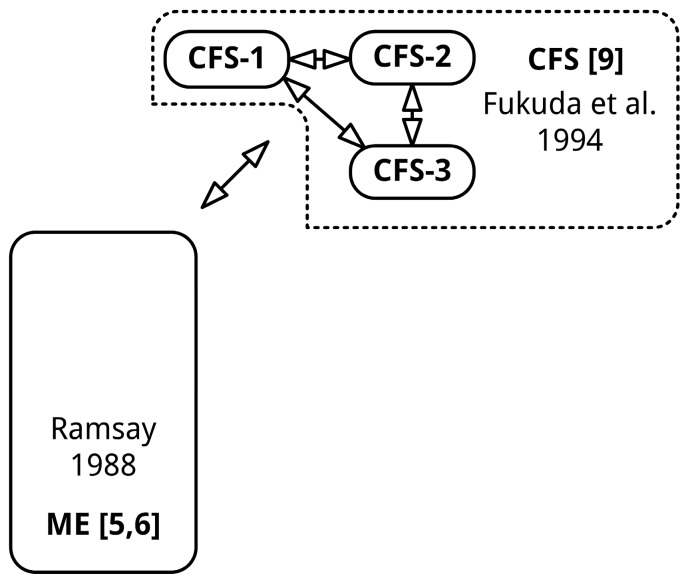
Proposed solution to resolve the diagnostic impasse. The sizes of the shapes do not reflect the absolute sizes of various patient (sub)populations.

Preferably diagnostic labels reflect the etiology, multiple sclerosis, or instigating agent, brucellosis. Although the name ME, especially the encephalomyelitis part of the name, is contested [29,46], some studies have found direct or indirect evidence of neuro-inflammation. Using recently proposed new criteria for ME [47], a recent study [48] observed neuro-inflammation in widespread brain areas in ME patients. Findings of a recent study into CFS [49] indicate “a markedly disturbed immune signature in the cerebrospinal fluid [...] consistent with immune activation in the central nervous system, and a shift toward an allergic or T helper type-2 pattern associated with autoimmunity.” Another study [43] found an increase in protein levels and number of white blood cells in 30% of the CFS [9] patients, found elevated IL-10 levels in patients with abnormal spinal fluids compared to patients with normal fluid or controls, and found higher levels of IL-8 in CFS patients with sudden, influenza-like onset. Therefore, there are indications of neuro-inflammation in CFS patients or subgroups. How many of these patients fulfilled the original criteria of ME [5,6] is unknown. Systemic exercise intolerance disease (SEID) does not reflect the clinical picture of ME. This abstract label captures the central symptom of the diagnostic criteria of SEID. Rather than choosing a new name, preserving the term ME would be sensible, since all “old” studies relate to ME, and the WHO has acknowledged ME as a neurological disease since 1969 [50]. If one would argue that neuro-inflammation is not (sufficiently) proven in ME yet, the eponymous Ramsay’s disease would by far be the most sensible alternative. As mentioned, the CFS [9] criteria define a heterogeneous group of people with chronic fatigue [10,11,12]. The polythetic nature of the definition of CFS (List 2) allows for 163 different combinations of symptoms. As mentioned, pattern analysis should be used to reveal symptom clusters/diseases currently classified as CFS. The “symptoms clusters” under the CFS “umbrella diagnosis” (“CFS-1,” “CFS-2,” *etc.*) should be named after the mandatory symptom(s) in each disorder until the exact etiology of that disorder is unraveled by future research (Figure 4).

## 5. Discussion

Due to the abstract and subjective nature of characteristic symptoms and the lack of clear etiologic explanations, ME and CFS are still surrounded by controversy. It is good to note a change of direction in the attitude towards ME/CFS. Indeed, “ME/CFS is a serious, chronic, complex systemic disease that often can profoundly affect the lives of patients” [14] with clear immunological, neurological, endocrine, autonomic and metabolic abnormalities [51,52]. ME/CFS is “a medical—not a psychiatric or psychological—illness” [52]. Green and colleagues [51] take a firm stand by posing that “both society and the medical profession have contributed to the disrespect and rejection experienced by patients with ME/CFS.” In order to improve the situation for patients, the IOM proposes to replace ME and CFS with SEID [14], with a key role for “exercise intolerance,” a highly characteristic feature of ME and CFS.

However, despite good intentions, introducing a new clinical entity SEID [14] does not resolve the most important methodological and diagnostic issues. Firstly, a new clinical entity cannot replace two distinct, partially overlapping, clinical entities. ME/CFS is a “hybrid diagnosis”: Only part of the CFS [9] patient population meet the more strict criteria for ME [5,6], while ME [5,6] patients not reporting fatigue or at least four of the “additional” symptoms do not meet the diagnosis CFS [9]. Secondly, due to their abstract and ill-defined nature of the diagnostic criteria of SEID, the proposal to employ self-report, instead of an objective assessment of typical symptoms, and the lack of exclusion criteria to preclude patients with other medical and psychiatric conditions, the diagnostic criteria of SEID seem to select an even more heterogeneous patient population of patients with chronic fatigue, thereby causing further scientific and diagnostic confusion, not reducing confusion. The position that “the new IOM case definition and algorithm [14] provide a starting place for future studies of diagnostic testing” [15] for ME and CFS is seriously open to question for the arguments given.

## 6. Conclusions

Replacing ME [5,6] and CFS [9] by a third clinical entity does not resolve the diagnostic and scientific impasse. SEID is based upon the invalid premise that ME and CFS are similar conditions, a thorough analysis of scientific literature that mainly relates to CFS, and consensus. As a logical consequence, SEID [14] does not capture the essence of ME [5,6]. Moreover the diagnostic criteria of SEID are not more restrictive than the CFS criteria [9] and also apply to people with other medical and psychological conditions. Self-report and subjective measures, as proposed by the IOM [14], are not adequate for diagnosis and assessment of the clinical status of patients in research.

To resolve the diagnostic impasse and to improve the quality of research, (a) the scientific community should acknowledge that much of the confusion originates from merging two clinical entities, ME and CFS, into a “hybrid diagnosis” (ME/CFS); (b) symptoms should be assessed by objective measures, not by self-report only; (c) pattern recognition methods should be used to establish the optional symptoms of ME [5,6], and to reveal “symptom clusters”/disorders covered by the “umbrella diagnosis” CFS, taking into account confounding variables, like onset, and duration of illness; and (d) diagnostic labels should preferably reflect the clinical picture.
